# Development of a Humanized VHH Based Recombinant Antibody Targeting Claudin 18.2 Positive Cancers

**DOI:** 10.3389/fimmu.2022.885424

**Published:** 2022-06-28

**Authors:** Weixiang Zhong, Yimin Lu, Zhe Ma, Yinjun He, Yongfeng Ding, Gaofeng Yao, Zhenxing Zhou, Jiali Dong, Yongliang Fang, Weiqin Jiang, Weilin Wang, Yanshan Huang

**Affiliations:** ^1^ Department of Pathology, The First Affiliated Hospital, School of Medicine, Zhejiang University, Hangzhou, China; ^2^ Department of Surgical Oncology, The First Affiliated Hospital, School of Medicine, Zhejiang University, Hangzhou, China; ^3^ Department of Innovative Drug Discovery and Development, Zhejiang Doer Biologics Co., Ltd., Hangzhou, China; ^4^ School of Medicine, Zhejiang University, Hangzhou, China; ^5^ Department of Medical Oncology, The First Affiliated Hospital, School of Medicine, Zhejiang University, Hangzhou, China; ^6^ Department of Hepatobiliary and Pancreatic Surgery, The Second Affiliated Hospital, School of Medicine, Zhejiang University, Hangzhou, China

**Keywords:** Claudin 18.2, VHH, gastric cancer, pancreatic cancer, ADCC, CDC

## Abstract

Claudin 18.2 (CLDN18.2), a tight junction (TJ) family protein controlling molecule exchange between cells, is frequently over-expressed in gastric cancer, pancreatic adenocarcinomas and in a fraction of non–small cell lung cancer cases. The tumor properties indicate that CLDN18.2 could be an attractive drug target for gastric and pancreatic cancers. In this study, we present effective strategies for developing anti-CLDN18.2 therapeutic candidates, based on variable domain of heavy chain of heavy chain antibodies (VHHs). CLDN18.2-specific VHHs were isolated by panning a phage display library from an *alpaca* immunized with a stable cell line highly expressing CLDN18.2. Humanized VHHs fused with human IgG1 Fc, as potential therapeutic candidates, exhibited desirable binding specificity and affinity to CLDN18.2. *In vitro* experiments showed that hu7v3-Fc was capable of eliciting both antibody-dependent cellular cytotoxicity (ADCC) and complement-dependent cytotoxicity (CDC) on CLDN18.2 positive tumor cells. In the mouse xenograft model, the anti-tumor efficacy of hu7v3-Fc was significantly more potent than Zolbetuximab, the benchmark anti-CLDN18.2 monoclonal antibody. Moreover, *in vivo* biodistribution using zirconium-89 (^89^Zr) labeled antibodies demonstrated that hu7v3-Fc (^89^Zr-hu7v3-Fc) exhibited a better tumor penetration and a faster tumor uptake than Zolbetuximab (^89^Zr-Zolbetuximab), which might be attributed to its smaller size and higher affinity. Taken together, anti-CDLN18.2 hu7v3-Fc is a promising therapeutic agent for human CLDN18.2 positive cancers. Furthermore, hu7v3 has emerged as a potential module for novel CLDN18.2 related therapeutics.

## Introduction

Gastric cancer (GC) and pancreatic cancer (PC), both accounted for 12.4% of all cancer mortalities in 2020 (1), are among the leading causes of cancer-related deaths worldwide. Due to insufficient early symptoms, patients with gastric cancer and pancreatic cancer are usually diagnosed at advanced stages with poor prognosis. Although various therapeutic approaches, such as chemotherapies, immunotherapies and targeted therapies, have been developed, the 5-year survival rates for patients with advanced GC and PC are still dismal (2, 3).

Claudin 18 (CLDN18) is a member of the claudin family with four transmembrane domains. CLDN18 has two extracellular loops, loop 1 and loop 2. CLDN18 has two splice variants in human, CLDN18.1 and CLDN18.2, which differ by 21 amino acids among the first 69 amino acids at the N-terminus but only 8 amino acids in the extracellular domain 1 (4). In normal healthy tissues, CLDN18.1 is strictly expressed on epithelial cells of lung tissue, while CLDN18.2 is confined to differentiated epithelial cells in stomach, such as mucous cells, parietal cells and chief cells (5, 6). However, CLDN18.2 is abnormally expressed in multiple cancers, including diffuse-type GC (7), PC (5, 8, 9), esophageal adenocarcinomas (10) and a small proportion of non–small cell lung cancer (11).

The variable domains of heavy chain of heavy chain antibodies (VHHs) represent the smallest naturally derived antigen-recognizing domains. Because of the significantly smaller in size than conventional monoclonal antibodies, VHHs could have a better tumor penetration and a faster tumor uptake than conventional monoclonal antibodies (12). Moreover, VHHs are highly stable and could be easily employed as building blocks for multiple formats of bi-specific antibodies and tri-specific antibodies with high affinity and avidity (13, 14). Thus, VHHs have certain advantages over conventional IgG in antibody drug development.

Here in this study, we present effective strategies for developing anti-CLDN18.2 VHH antibodies with high affinity and specificity. hu7v3-Fc, one of the humanized candidates fused with human IgG1 Fc, showed antibody-dependent cellular cytotoxicity (ADCC) and complement-dependent cytotoxicity (CDC) on CLDN18.2 positive tumor cells *in vitro*. Meanwhile in the mouse xenograft model, hu7v3-Fc demonstrated strong efficacy of anti-tumor and nuclides targeted delivery ability, indicating that it is a promising therapeutic agent for human CLDN18.2 positive cancers.

## Materials and Methods

### Cell Lines and Culture Conditions

CHO-K1, CHO-CLDN18.1-GFP (CLDN18.1 and GFP co-expressing) and CHO-CLDN18.2-GFP (CLDN18.2 and GFP co-expressing) cells were cultured in CD02 medium (Quacell) at 37°C in a 5% CO_2_ incubator with constant agitation at 120 rpm. ADCC bioassay effector cells were maintained in 90% RPMI1640 medium (BasalMedia) with L-glutamine and 10% fetal bovine serum (FBS, ExcellBio), 100 µg/mL hygromycin (Invivogen), 250 µg/mL G-418 sulfate solution (Invivogen), sodium pyruvate (1 mM, Gibco) and MEM non-essential amino acids (0.1 mM, Gibco), at 37°C in a humid incubator with 5% CO_2_. SNU-620 (human gastric cancer cell line with endogenous CLDN18.2 expression) and NUGC4-CLDN18.2 (human gastric cancer cell line with exogenous CLDN18.2 expression) cells were grown in RPMI1640 medium supplemented with 10% FBS. MIA PaCa-2-CLDN18.2 cells (human pancreatic cancer cell line with exogenous CLDN18.2 expression) were grown in DMEM high glucose medium supplemented with 10% FBS.

### Phage Library Construction

A healthy female *alpaca* was immunized with 1.0 × 10^7^ CHO-CLDN18.2-GFP cells, with Freund’s adjuvant (Sigma) as an immunopotentiator. A total of three immunizations were performed with an interval of 21 days between each immunization. Next, 30 mL of *alpaca* whole blood was harvested in a vacuum blood collection tube a week after the final immunization. Lymphocytes were then isolated using separation medium (Ficoll-Paque Plus, Sigma). Extracted total RNA (Trizol, Ambion) was used as a template for synthesizing cDNA by reverse transcription (Super Scrip III First Strain, Invitrogen). Synthesized cDNA was used as a template for the NEST polymerase chain reaction (PCR) to amplify the VHH sequences. The amplification primers for the first round of PCR were 5′-CTTGGTGGTCCTGGCTGC-3′ and 5′-ggtacgtgctgttgaactgttcc-3′. The products from the first round of PCR were used as a template for the second round. The forward and reverse primers used in the second round PCR were 5′-CATGCCATGACTGTGGCCCAGGCGGCCCAGKTGCAGCTCGTGGAGTC-3′ and 5′-CATGCCATGACTCGCGGCCGGCCTGGCCGTCTTGTGGTTTTGGTGTCTTGGG-3′, respectively. Amplified VHH fragment products were collected with a gel extraction kit (BioMIGA). Purified fragments were digested by *Sfi*I (NEB) and then ligated to phagemid pCom3xss (Addgene, #63890). The ligation products were transformed into an *Escherichia coli–*competent strain, ER2738, by electroporation.

### Bio-panning of CLDN18.2 VHH

The cloned VHH repertoire was expressed at the tip of M13 virions with the assistance of M13KO7 helper phage (NEB). CHO-K1 and CHO-CLDN18.2-GFP cells were used in the first round of bio-panning to eliminate non-specific phage binders and enrich positive phages. Briefly, 4.5 × 10^7^ CHO-K1 cells were washed with phosphate-buffered saline (PBS) before being blocked with 2% milk powder. A phage library (approximately 5.7 × 10^11^ plaque-forming units) was added to the blocked CHO-K1 cells and rotated at 4°C for 1 h. Cells were pelleted to collect the supernatant which was then added to the blocked CHO-CLDN18.2-GFP cells (1.5 × 10^7^ cells) and incubated at 4°C for 1 h. To elute bound phages, cells were pelleted and washed before being resuspended in 1 mL of 0.1 M Glycine-HCl (pH 2.2) with 1 mg/mL bovine serum albumin (BSA, Sango Biotechnology). After centrifugation, the supernatant was collected and neutralized with 1 M Tris-HCl (pH 8.0). The solution containing the enriched phage particles with CLDN18.2-specific VHH was used to infect 5 mL of logarithmic phase ER2738. Negative screening (by CHO-K1 cells and CHO-CLDN18.1-GFP cells) and positive screening (by CHO-CLDN18.2-GFP cells) was performed for another two rounds of panning before anti-CLDN18.2 phages were enriched. Eighty clones were randomly picked and cultured in a deep 96-well plate at 37°C. Cultures were then infected with M13KO7 helper phages to rescue and express the VHH at the tip of phage particles.

The cellular enzyme-linked immunosorbent assay was used to determine positive clones. The supernatant from the phage clones was diluted with 3% BSA. 50 µL of diluted phages were added to 96-well microtiter plates containing 50 µL of BSA-blocked CHO-K1, CHO-CLDN18.1-GFP, and CHO-CLDN18.2-GFP cells (5 × 10^5^ cells each well). Mouse anti-M13 phage-horseradish peroxidase conjugate (Sinobiological) was used as a detection antibody, and tetramethylbenzidine was used as a visualizing agent. The reactions were terminated by 2 M H_2_SO_4_, and absorption was measured at 450 nm by a VersaMax microplate reader (Molecular Devices). Eighteen putative positive clones were further sequenced and eight unique clones were obtained finally.

### Humanization, Expression and Purification

Anti-CLDN18.2 VHHs were humanized as described previously (15). Briefly, complementarity determining regions (CDR1, CDR2 and CDR3) of the NbBcII10*_FGLA_* (PDB:3EAK) scaffold were replaced with the corresponding region of anti-CLDN18.2 VHHs, according to the International ImMunoGeneTics information system (IMGT) amino acid numbering. Furthermore, crucial amino acid residues of FR2 were substituted to mimic the human DP-47 reference sequence, in order to improve the physicochemical properties.

The DNA fragment coding for the human IgG1 Fc (16), containing the full hinge region (EPKSSDKTHTCPPCP), was fused to the DNA fragment coding for the full length of VHH and cloned into the pcDNA3.1 expression plasmid. VHH-Fc encoding plasmids were introduced into HEK293F cells with polyethyleneimine (Polysciences, Inc.). The transfected cells were cultured for 7 days and supernatant was harvested. The desired products were further purified with Protein A affinity column (BestChrom). The benchmark Zolbetuximab were expressed and purified following the patent US9751934B2.

### Immunohistochemistry

The frozen tissue section slides were fixed in acetone for 15 minutes and subsequently washed by PBS. Endogenous peroxidases and non-specific binding were blocked by incubating with 3% hydrogen peroxide for 10 minutes and with 3% BSA, respectively. Slides were incubated with primary antibodies for 30 minutes at room temperature, followed by a 30-minute incubation with horseradish peroxidase-conjugated goat anti human IgG antibodies (Thermofisher). Antibody binding was visualized by incubating with the peroxidase substrate 3,3’-diaminobenzidine (DAB, GeneTech) for 5 minutes. After counterstaining with hematoxylin (Sangon Biotechnology), tissue sections were analyzed using light microscopy (Nikon).

### Antibody-Dependent Cellular Cytotoxicity Assay

The reporter-based surrogate ADCC bioassay was carried out with Jurkat cells, engineered to express FcγRIIIa and an NFAT response element driving expression of firefly luciferase, as effector cells. Briefly, 1.5 × 10^5^ ADCC bioassay effector cells (E) were mixed with 3 × 10^4^ CHO-CLDN18.2-GFP target cells (T) to make the final E:T = 5:1, for a final volume of 50 µL in a U-bottom plate. Test antibodies were then serially diluted into a final volume of 25 µL and added to the assay system before placed in a CO_2_ incubator for 20 h. Finally, 75 µL of BIO-Glo Luciferase Assay Reagent (Promega) was added to the assay system to measure luminescence using a SpectraMax i3 (Molecular Devices).

Peripheral blood mononuclear cells (PBMCs) were used as effector cells and SNU-620 cells were used as target cells in PBMC-based assays. Briefly, 5.0 × 10^5^ PBMCs were mixed with 2 × 10^4^ SNU-620 cells to make the final E:T ratio 25:1, for a final volume of 100 µL in a U-bottom plate. Test antibodies were then serially diluted into a final volume of 50 µL and added to the assay system before incubated for 20 h. The culture supernatants were collected and applied to LDH release assay (Promega).

### Complement-Dependent Cytotoxicity Assay

Complement-dependent cytotoxicity was assessed by flow cytometry. Briefly, CHO-CLDN18.2-GFP and NUGC-CLDN18.2 were harvested and resuspended in Opti-MEM medium (Thermofisher). Cell density was adjusted to 1 × 10^6^ cells/ml and 50 μl of cells was seeded into each well. 50 μl of test antibodies at various concentration and 50 μl of human AB serum (15%, Schbio) used as a source of the complement were added into each well. The plate was placed at 37°C in a 5% CO_2_ incubator for 2 hours. Thereafter, add 50 μl of propidium iodide (PI, Thermofisher) into each well and analyze the cells using a flow cytometer immediately.

### Xenograft Tumor Model

To establish human gastric cancer or pancreatic cancer xenograft models, six-eight weeks old female CB17 SCID mice (Vital River Laboratory) were subcutaneously injected with 1×10^7^ SNU-620 cells or 1×10^7^ MIA PaCa-2-CLDN18.2 cells, mixed with Matrigel Matrix (Corning) with a ratio of 1:1. The mice were randomly divided into five groups: PBS as a vehicle control, 5.7 mg/kg Zolbetuximab as a positive control, 0.3 mg/kg, 1.0 mg/kg and 3.0 mg/kg hu7v3-Fc as the experimental groups, when the tumor volumes reached 170 mm^3^ or 236 mm^3^. Test antibodies were given by intraperitoneal injection three times per week (TIW) for five weeks.

Tumor volume were measured twice per week with a vernier caliper and determined according to the following equation: Tumor volume (mm^3^) = 1/2 length × width^2^. After the mice were sacrificed, the tumors were dissected and photographed.

### *In vivo* Biodistribution Studies

*In vivo* biodistribution studies were performed using a SUN-620 xenograft mouse model to compare the tumor update between hu7v3-Fc and Zolbetuximab. Both hu7v3-Fc and Zolbetuximab were radiolabeled with zirconium-89 (^89^Zr), formulated, and administered to mouse respectively (17). Mice were anesthetized and sacrificed at 4, 24, 48, 72, 96, 120 h, respectively. The amounts of radioactivity in interest tissues (Brain, heart, liver, spleen, lung, kidney, muscle, bone and the tumor) were measured with a gamma counter. Radioactivity uptake was calculated as the percentage of the injected dose per gram of tissue (%ID/g).

### Statistical Analysis

All statistical analyses were performed with Graphpad Prism 6 software. Experimental data were expressed as mean ± SD or mean ± SEM. Two-group comparisons were analyzed by Student’s *t* test. *p* < 0.05 was considered significant.

## Results

### Library Construction and Bio-panning

As a membrane protein with four transmembrane domains, it is challenging to prepare purified recombinant CLDN18.2 protein in its native conformation. In this study, we constructed a phage display library after immunization of an *alpaca* with CHO-CLDN18.2-GFP cells, which overexpress the full length of CLDN18.2. VHH gene segments were cloned into a phagemid vector, resulting in a phage library of 1.23 × 10^8^ size.

**Figure 1:**
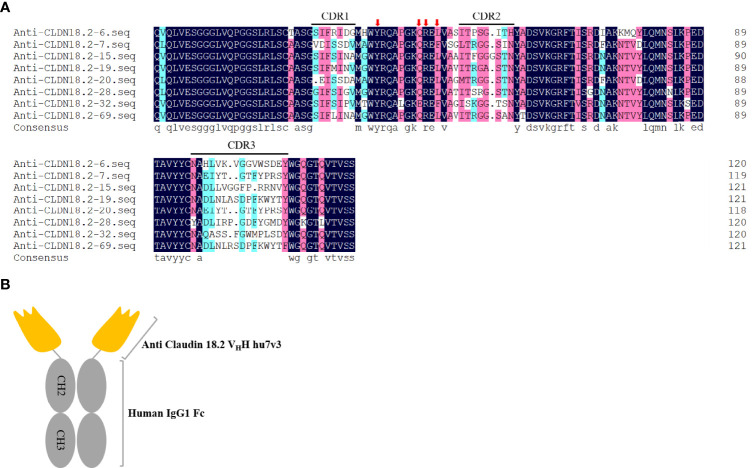
Isolation of anti-CLDN18.2 VHH variants. **(A)** Amino acid sequences of anti-CLDN18.2 VHHs. Sequences are presented using one-letter amino acid abbreviations. DNAMAN was used to generate the alignment. Complementarity determining regions (CDRs) are shown. Four hydrophilic amino acids (Tyr42, Glu/Gln49, Arg50 and Leu/Phe52) critical for humanization are indicated with red arrows. **(B)** Schematic composition of hu7v3-Fc structure in this study. Anti CLDN18.2 VHH hu7v3 is indicated in orange, and the human IgG1 Fc domain including the hinge, CH2 and CH3 domains is indicated in gray.

After three rounds of bio-panning, eighty clones were randomly picked for CLDN18.2-binding test, and 18 potential positive clones were identified. After sequence analysis, eight unique sequences were successfully obtained. Compared with conventional VH region, of which the framework region 2 (FR2) has highly conserved hydrophobic amino acids (Val42, Gly49, Leu50 and Trp52) (18), the four amino residues are substituted for more hydrophilic amino acids (Tyr42, Glu/Gln49, Arg50 and Leu/Phe52) in our isolated VHHs (**Figure 1A**). Meanwhile, all the clones have longer CDR3 loops with 14~16 amino acids, which is a typical feature of VHHs (19). Using the anti-CLDN18.2-7 as an example, all the VHHs were further humanized as described previously (15). To increase stability, solubility, and affinity, crucial amino acid residues were substituted to obtain an optimum version of humanized VHH, anti-CLDN18.2-hu7v3 (**Supplementary Figure S1**). The humanized VHH was fused to the Fc domain of human IgG1 to produce a homodimer chimeric protein, anti-CLDN18.2-hu7v3-Fc (simplified as hu7v3-Fc), capable of bivalent binding, ADCC and CDC activities (**Figure 1B**). Three of the Fc fusion proteins, hu7v3-Fc, hu28v3-Fc and hu69v3-Fc, showed higher affinity to CLDN18.2 than others (**Supplementary Figure S2**). As hu28v3-Fc and hu69v3-Fc showed lower solubility and stronger aggregation (data not shown), we chose hu7v3-Fc as a leading drug candidate.

### Characterization of hu7v3-Fc

**Figure 2:**
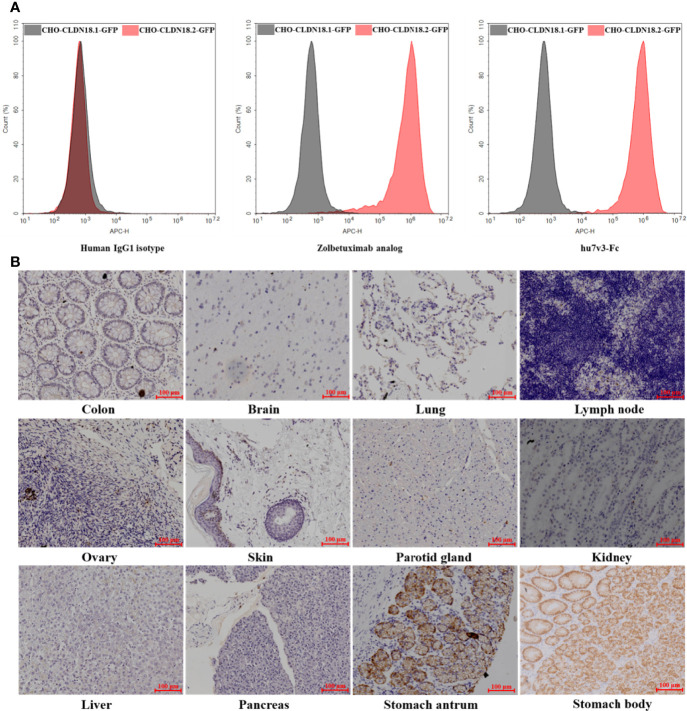
Characterization of anti-CLDN18.2 hu7v3-Fc. **(A)** Flow cytometric analysis to determine the specificity of hu7v3-Fc to CLDN18.2. Zolbetuximab was employed as the positive control antibody, and human IgG1 isotype as the negative control antibody. CLDN18.1 and CLDN18.2 was co-expressed with GFP in CHO-CLDN18.1-GFP and CHO-CLDN18.2-GFP cells, respectively. **(B)** IHC analysis to determine the specificity of hu7v3-Fc to CLDN18.2. Frozen sections of colon, brain, lung, lymph node, ovary, skin parotid gland, kidney, liver, pancreas, stomach antrum and stomach body were obtained from healthy tissue in The First Affiliated Hospital of Zhejiang University.

The specificity of hu7v3-Fc to recognize CLDN18.2 membrane protein was evaluated. In flow cytometry assays, the Zolbetuximab (benchmark) and human IgG1 isotype were employed as a positive control and a negative control, respectively. As shown in **Figure 2A**, MFI signals of hu7v3 and Zolbetuximab on CHO-CLDN18.2-GFP cells were significantly higher than that on CHO-CLDN18.1 cells, indicating an excellent specificity of hu7v3 to CLDN18.2 positive cells.

Because the expression of CLDN18.2 is strictly confined to the differentiated epithelial cells of the gastric mucosa, the binding specificity of hu7v3-Fc was also assessed with IHC. As demonstrated in **Figure 2B** and **Supplementary Figure S3**, only stomach antrum and stomach body tissue specimens showed strong positive staining for hu7v3-Fc, whereas other tissue specimens did not react with hu7v3-Fc. The binding affinity to CLDN18.2 was furtherly analyzed. As calculated from dose-responsive curves, hu7v3-Fc, with a *K_D_* value of 1.32 × 10^-9^ M, shows a higher affinity to CLDN18.2 than Zolbetuximab (**Supplementary Figure S4**).

### ADCC and CDC Efficacy of hu7v3-Fc

**Figure 3:**
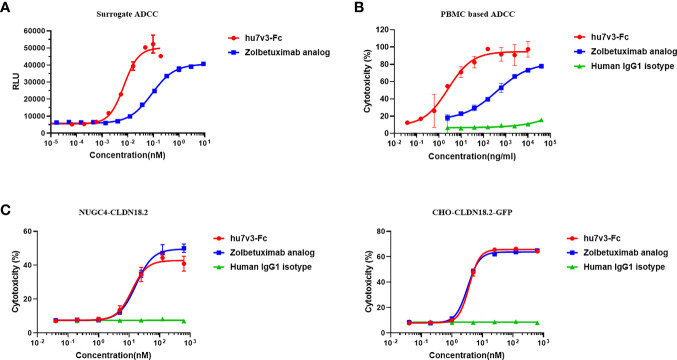
Cytotoxicity analysis of hu7v3-Fc. **(A)** The surrogate ADCC analysis of hu7v3-Fc. Jurkat cells, engineered to express FcγRIIIa and NFAT response element-driven firefly luciferase were used as the effector cells, and CHO-CLDN18.2-GFP cells were used as target cells. The effector to target ratio was 5:1. **(B)** PBMC based ADCC analysis of hu7v3-Fc. Human PBMCs isolated form whole blood were used as the effector cells, and SNU-620 were used as the target cells. The effector to target ratio was equal to 25:1. **(C)** CDC analysis of hu7v3-Fc. Human AB serum was used as a source of the complement, with NUGC4-CLDN18.2 (left) cells and CHO-CLDN18.2-GFP (right) cells as the target cells, respectively.

To determine the ability of hu7v3-Fc to trigger ADCC, reporter-based surrogate assay was performed, using engineered Jurkat cells expressing FcγRIIIa and NFAT response element-driven firefly luciferase as effector cells. PBMC-based LDH release assay was also performed. In the surrogate assay, the ADCC of hu7v3-Fc (EC_50_ = 0.007 nM) was 12.4-fold greater than that of Zolbetuximab (EC_50_ = 0.087 nM) against CHO-CLDN18.2-GFPs (**Figure 3A**). In the PBMC-based assay, the gastric cell line SNU-620 cells which endogenously express CLDN18.2 were used the target cells. The activity of hu7v3-Fc (EC_50_ = 2.583 ng/ml) to mediate ADCC was 82.7-fold higher than that of Zolbetuximab (EC_50 =_ 397.0 ng/ml), while the human IgG1 isotype failed to produce an obvious dose-dependent signal curve (**Figure 3B**), indicating the CLDN18.2 specific mechanism of action of hu7v3-Fc.

The CDC capacity of hu7v3-Fc was assessed using human AB serum as a source of complement and NUGC4-CLDN18.2 and CHO-CLDN18.2-GFP, both over-express CLDN18.2 (**Supplementary Figure S5A**), as target cells. Of note, flow cytometry analysis demonstrated that hu7v3-Fc and Zolbetuximab exhibited similar CDC activities on both target cells, while the IgG1 isotype control showed no obvious CDC activity (**Figure 3C**). Although hu7v3 opsonized cells are better at binding to C1q, this binding does not translate directly to a higher CDC activity (**Supplementary Figure S5.B**).

### Antitumor Activity of hu7v3-Fc *in vivo*

**Figure 4:**
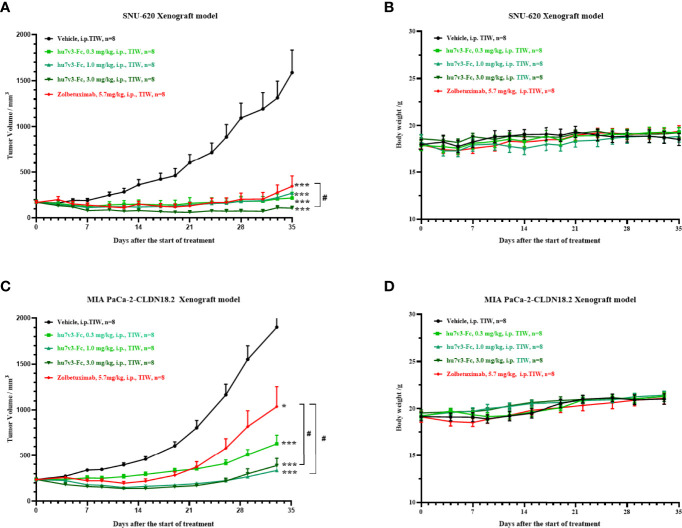
*In vivo* tumor inhibition efficacy of anti-CLDN18.2 7v3-Fc. **(A, C)** Tumor growth curves of SNU-620 and MIA PaCa-2-CLDN18.2 bearing mice injected with different formulations, respectively. **(B, D)** Body weight changes of SNU-620 and MIA PaCa-2-CLDN18.2 bearing mice injected with different formulations, respectively.Data are shown as mean ± SEM, n = 8, *p < 0.05, ***p < 0.001 (vs Vehicle); ^#^p< 0.05 (vs Zolbetuximab).

Next, we evaluated the antitumor effect of hu7v3-Fc using a human gastric cancer cell line SNU-620 cells xenograft model. The results indicated that SNU-620 tumor growth was significantly suppressed in mice even treated with low dosage of hu7v3-Fc (0.3 mg/kg), compared with PBS-treated group of mice. Meanwhile, the inhibitory effect of 3 mg/kg hu7v3-Fc on tumor growth was stronger than that of equal molar dosage (5.7 mg/kg) of Zolbetuximab (tumor growth inhibition (TGI): 104.5% vs 84.5%, p < 0.05) (**Figure 4A** and **Supplementary Table 1**).

A human pancreatic cancer cell line MIA PaCa-2-CLDN cells derived xenograft model was also created to assess the antitumor efficacy of hu7v3-Fc. As shown in **Figure 4C** and **Supplementary Table 2**, all the dosages of hu7v3-Fc (0.3 mg/kg, 1.0 mg/kg, 3.0 mg/kg) have stronger anti-tumor effects than Zolbetuximab (5.7 mg/kg). There were no significant differences in the body weight between the five groups (**Figures 4B, D**), suggesting that hu7v3-Fc is a safe and promising therapeutic candidate.

### Biodistribution Studies

**Figure 5:**
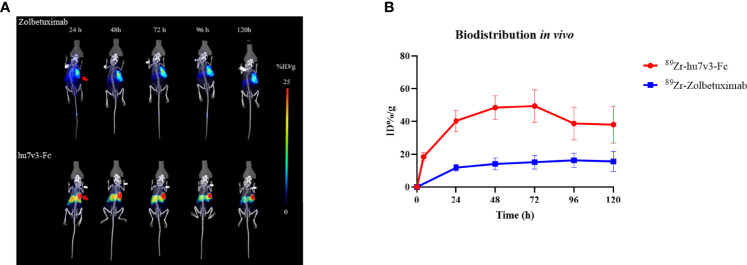
*In vivo* tumor location of ^89^Zr labeled 7v3-Fc. **(A)** ^89^Zr PET-CT imaging in mice bearing subcutaneously SNU-620 tumors showed specific tumor location. Red arrowheads indicate the location of tumors. **(B)** Time-radioactivity curves derived from PET-CT imaging after injection with ^89^Zr labeled hu7v3-Fc and Zolbetuximab. Data are displayed with Mean ± SD (n = 3).

Biodistribution studies with ^89^Zr-hu7v3-Fc and ^89^Zr-Zolbetuximab were investigated in a SUN-620 subcutaneous xenograft model. *In vivo* biodistribution at 4, 24, 48, 72, 96, 120 h post-injection was showed in **Figures 5A, B**. Compared with ^89^Zr-Zolbetuximab, ^89^Zr-hu7v3-Fc showed faster tumor uptake and better tumor penetration at all time points, and its accumulation in tumor tissue reached a plateau (49.43 ± 9.86 ID%/g) at 72 h. Meanwhile, ^89^Zr-hu7v3-Fc revealed higher tumor-to-muscle ratio and comparable tumor-to-liver ratio to ^89^Zr-Zolbetuximab (**Supplementary Figure S6**).

## Discussion

Globally, Gastric cancer is the 4^th^ most common malignancy and the 2^nd^ leading cause of cancer mortality (20, 21); while pancreatic cancer is the 12^th^ most common malignant disease and the 7th leading cause of cancer death (22). Even with effective early diagnosis, many patients are diagnosed with advanced or metastatic disease. Among the patients with advanced or metastatic stage, the survival outcomes are still dismal; the 5-year survival rate is less 20% for gastric cancer (23) and is only 2% for pancreatic cancer (24). Therefore, there are urgent unmet clinical needs for both of gastric cancer and pancreatic cancer.

In normal tissue, Claudin 18.2 is strictly expressed in gastric mucosa. However, the expression of CLDN18.2 is frequently ectopic activated in a diversity of human cancers (4), such as gastric cancer and pancreatic cancer. These characteristics suggest CLDN18.2 can be an ideal molecule for targeted therapy. Up to now, there were no approved antibody drugs against Claudin 18.2. Zolbetuximab, a chimeric IgG1 monoclonal antibody, is the first-in-class antibody drug under clinical development that specifically binds to CLDN18.2 and mediates cell death by triggering ADCC and CDC activities (25). Phase II clinical trial revealed that, for the overall population, both progression-free survival (PFS) and overall survival (OS) were significantly improved in zolbetuximab combined with EOX group, as compared with those in EOX alone group (median PFS: 7.5 months vs 5.3 months, p < 0.0005; median OS: 13.0 months vs 8.3 months, p < 0.0005) (26). The most adverse events related to therapy include grade 1-2 of nausea, vomiting, neutropenia and anaemia, indicating the safety of CLDN18.2-targeting strategy in gastric cancer. Meanwhile, preclinical studies demonstrated that zolbetuximab mediated antitumor activity *via* inducing ADCC and CDC in pancreatic cancer models (27). Other alternative CLDN18.2-targeting therapeutic agents, such as CAR-T (NCT03890198, NCT03159819), bispecific antibody (NCT04260191) and ADC (NCT05009966, NCT04805307), are also ongoing.

Since the discovery of heavy chain only antibodies from camelid family in 1993, the application field of the VHHs has been rapidly growing. Being significantly smaller in size than the conventional monoclonal antibodies, VHHs usually have a better tumor penetration and a faster tumor uptake than conventional monoclonal antibody, as we demonstrated in **Figure 5**. This is a big advantage of VHHs compared to conventional monoclonal antibody drugs. Moreover, their longer CDR3 loops allows them to access the buried or hidden epitopes in proteins, such as the active sites of enzymes (28). Furthermore, VHHs can be building blocks for multiple bi-specific antibodies and tri-specific antibodies which can target multiple antigens and are of properties that are not offered by combination of two or three monoclonal antibodies (13, 14). Caplacizumab, the first VHH-based medicine, has been approved by FDA for adults with acquired thrombotic thrombocytopenic purpura (aTTP) in 2019 (29). VHHs labeled with different isotopes or fluorophores have been applied for tumor imaging (30), such as epidermal growth factor receptors (EGFRs) positive cancers. VHHs can be expressed as multi-specific formats simultaneously targeting multiple proteins with high affinity and their serum half-life can be prolonged by PEGylation, by fusion with long-lived serum albumin or IgG-Fc (31); or by fusion with an affinity reagent that recognizes and binds such long-lived serum proteins (32). Additionally, fusions of targeting VHHs to human IgG1 Fc can be used to recruit effector functions of ADCC and CDC (33). Altogether, development of VHH-based therapeutic agents is now in the ascendant with broad medical needs.

The use of synthetic peptide conjugated with carrier protein such as keyhole limpet hemocyanin (KLH) or BSA is the most popular strategy to generate antibodies (34). However, anti-peptides antibodies usually fail to recognize the native-state structure of membrane protein on the living cells. As most currently available VHHs recognize conformational epitopes rather than linear peptides (35), in our study, CHO-K1 cells engineered to express CLDN18.2 were used as immunogen in an *alpaca*. By cloning the VHH repertoire from the blood lymphocytes, expressing this repertoire on phages and bio-panning phages on CLDN18.2 expressing cells, eight CLDN18.2 positive VHHs were retrieved.

To improve affinity, circulation half-life, capacity to mediate anti-tumor cytotoxicity such as ADCC and CDC, the humanized anti-CLDN18.2 VHH 7v3 was fused to human IgG1 Fc to generate a bivalent fusion protein hu7v3-Fc. Flow cytometric assays and IHC staining demonstrated that hu7v3-Fc binds to CLDN18.2 with high affinity and specificity. In the cytotoxic assays, hu7v3-Fc was stronger than Zolbetuximab in the capacity to induce ADCC. Although hu7v3-Fc opsonized cells revealed a higher affinity to C1q, it mediated a CDC potency comparable to that by Zolbetuximab. The uncorrelation between C1q binding ability and CDC potency may stem from the nature of the assay methods. As the C1q binding assay was subjected to multiple cycles of washing step, Zolbetuximab because of its lower affinity is susceptible to be washed away form cell surface. In the CDC assay, Zolbetuximab could retained on the cell surface as no washing steps. Additionally, the possibility that hu7v3 and Zolbetuximab recognize different epitopes, thus affecting the CDC activity, cannot be ruled out.

In the mouse xenograft models, hu7v3-Fc was shown to be an promising therapeutic agent targeting CLDN18.2. In addition, hu7v3-Fc (around 75 kDa) is considerably smaller than Zolbetuximab (around 150 kDa). As a result, hu7v3-Fc exhibited faster tumor uptake, better tumor penetration and higher tumor-to-muscle ratio than Zolbetuximab, which may translate to improved therapeutic efficacy in cancer patients. It is worth noting that Zolbetuximab was prepared according to the patent US9751934B, any differences in production cell line, culture conditions and formulation, may attribute to the efficacy of the final product. Additionally, we performed long term toxicity study of hu7v3-Fc in SD rats and cynomolgus monkeys (data not shown), and found gastric atrophy/mixed cell inflammation/epithelial hyperplasia in hu7v3 treated animals during the dosing phase. However, a trend of recovery was observed at the end of the recovery period.

In summary, by whole cell immunization of an *alpaca*, a series of VHHs binding to CLDN18.2 with high specificity and affinity were discovered. The lead molecule, anti-CLDN18.2 hu7v3-Fc, demonstrated enhanced *in vitro* and *in vivo* anti-tumor activities, compared to the benchmark Zolbetuximab in CLDN18.2-positive gastric and pancreatic tumor cells. Additionally, these VHHs-based CAR-T and bispecific antibody for cancer therapy are under ongoing development. Future study will be conducted on incorporation of combination therapies, including chemotherapy, checkpoint blockade and cytokine therapies to further improve treatment of CLDN18.2 positive solid tumors.

## Data Availability Statement

The original contributions presented in the study are publicly available. This data can be found here: http://getentry.ddbj.nig.ac.jp/ under the accession numbers LC705046 ~LC705053.

## Ethics Statement

The studies involving human participants were reviewed and approved by Clinical Research Ethics Commit of the First Affiliated Hospital, College of Medicine, Zhejiang University. The patients/participants provided their written informed consent to participate in this study. The animal study was reviewed and approved by Animal Ethics Committee of Zhejiang Academy of Medical Science and Hangzhou Normal University.

## Author Contributions

WZ and YL designed the study, analyzed the data, performed the experiments and wrote the manuscript. ZM, GY, ZZ and JD performed the immunization and isolation of VHH. YH and YD performed the IHC. WJ and YF supervised the project. Funding acquisition was carried out by WW. WW and YSH designed the study, supervised the project and wrote the manuscript. All authors contributed to the article and approved the submitted version. 

## Funding

The work was supported by the National Natural Science Foundation of China (81802350).

## Conflict of Interest

ZM, GY, ZZ, JD, YF, and YSH are current or former employees of Zhejiang Doer Biologics Co., Ltd. YSH is a shareholder of Zhejiang Doer Biologics Co., Ltd.

The remaining authors declare that the research was conducted in the absence of any commercial or financial relationships that could be construed as a potential conflict of interest.

## Publisher’s Note

All claims expressed in this article are solely those of the authors and do not necessarily represent those of their affiliated organizations, or those of the publisher, the editors and the reviewers. Any product that may be evaluated in this article, or claim that may be made by its manufacturer, is not guaranteed or endorsed by the publisher.
