# Supplementary figures and images for: Development of a Humanized VHH Based Recombinant Antibody Targeting Claudin 18.2 Positive Cancers
Source: Front Immunol. 2022 Jun 28;13:885424. doi: 10.3389/fimmu.2022.885424 (PMC9273722; doi:10.3389/fimmu.2022.885424)

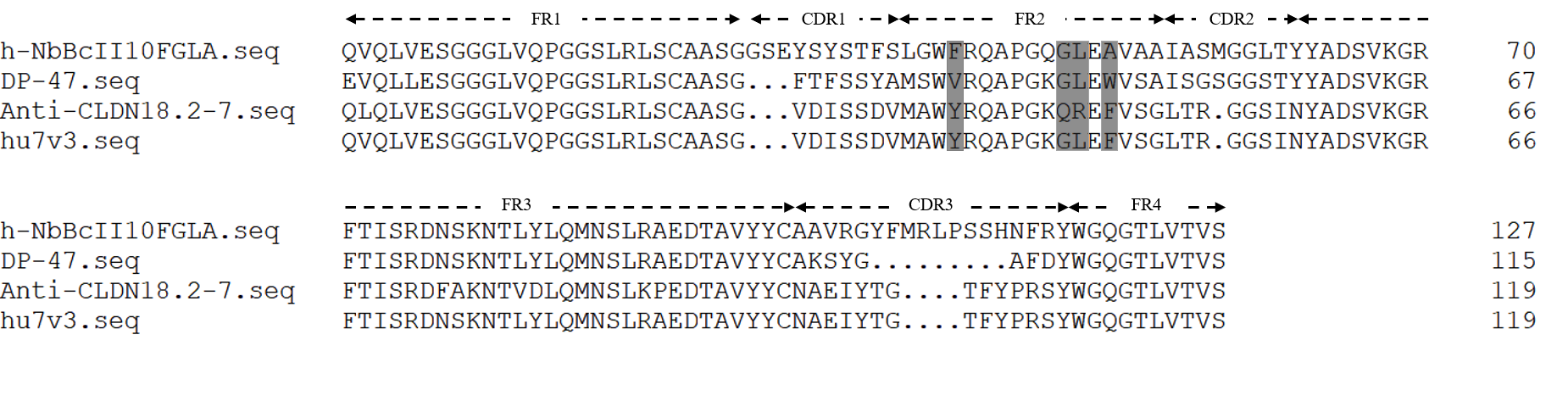

Supplement: Supplementary file 1 [file Image_1.tif]

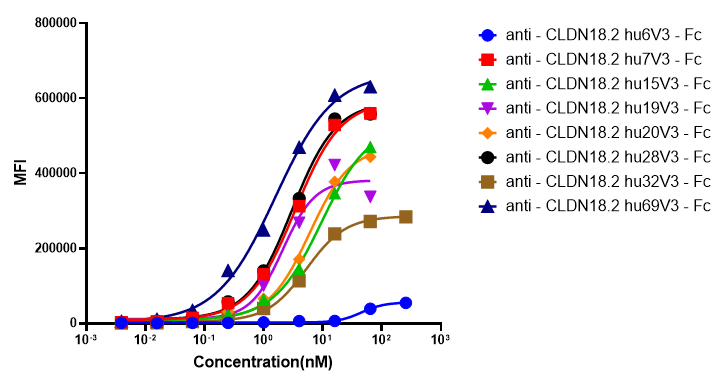

Supplement: Supplementary file 2 [file Image_2.tif]

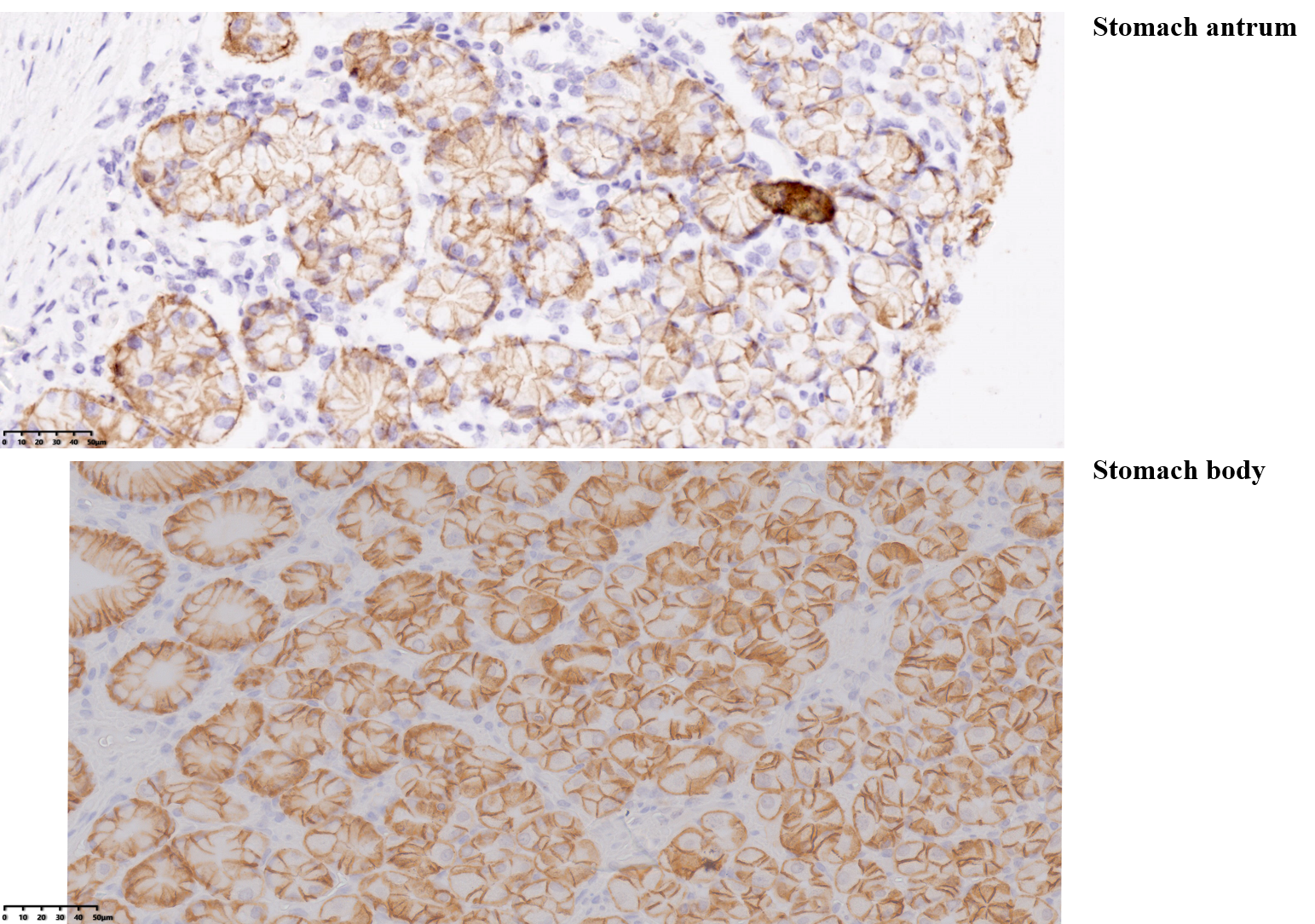

Supplement: Supplementary file 3 [file Image_3.tif]

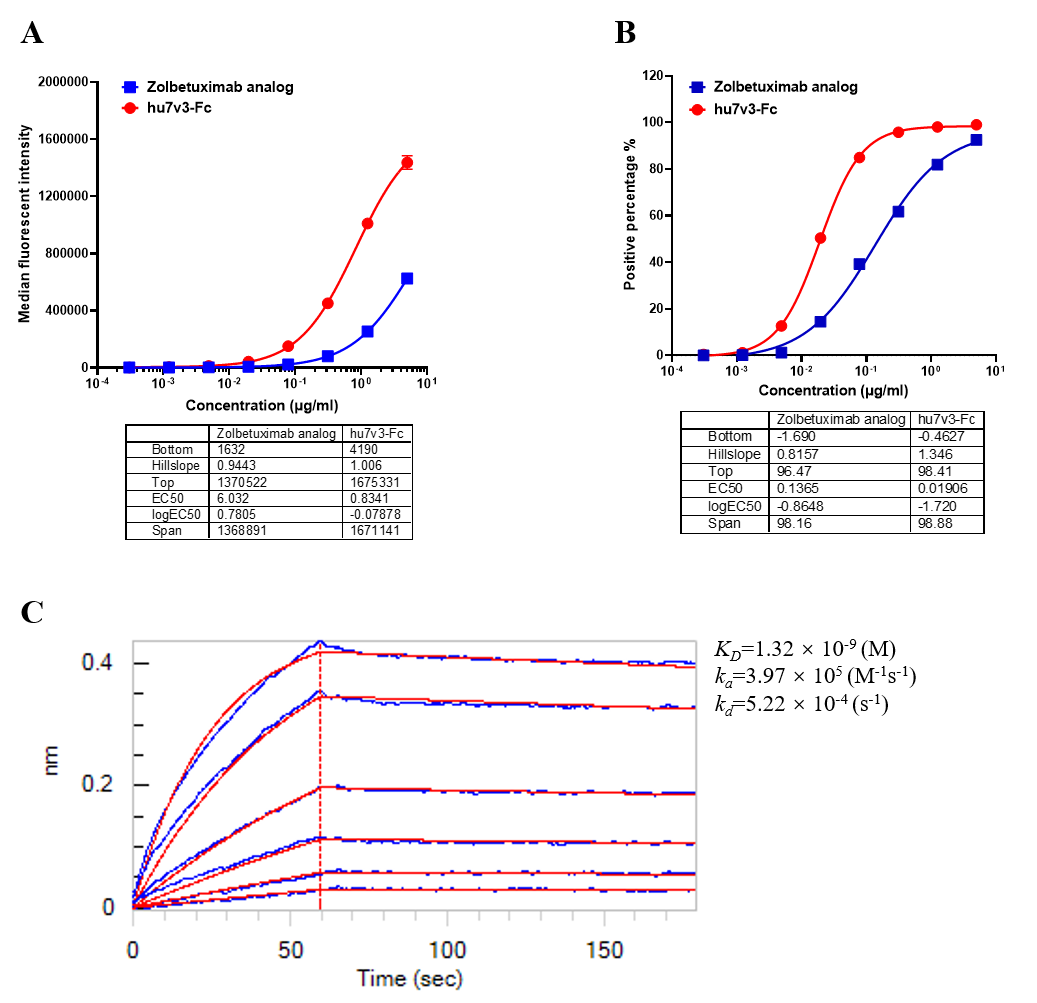

Supplement: Supplementary file 4 [file Image_4.tif]

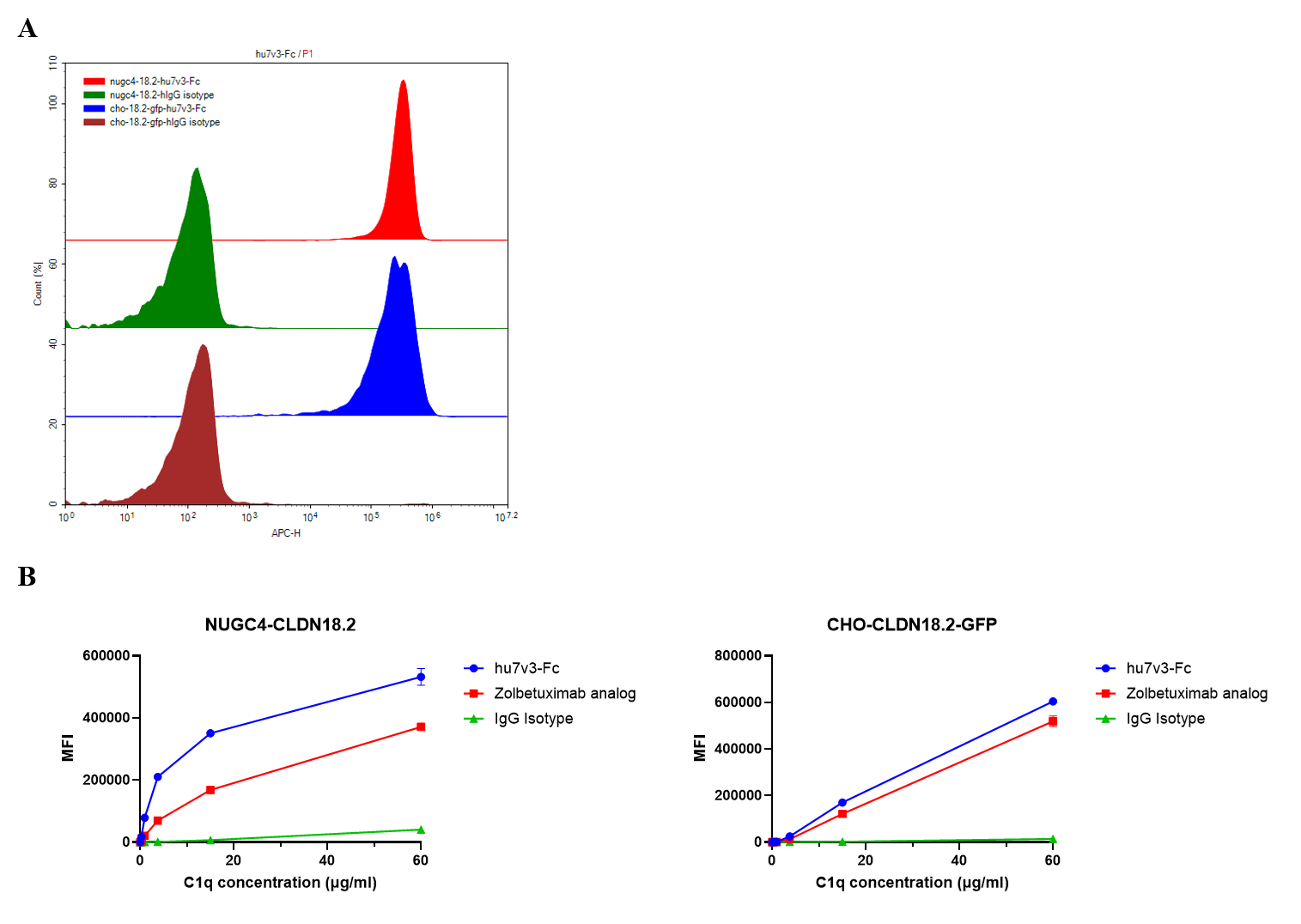

Supplement: Supplementary file 5 [file Image_5.tif]

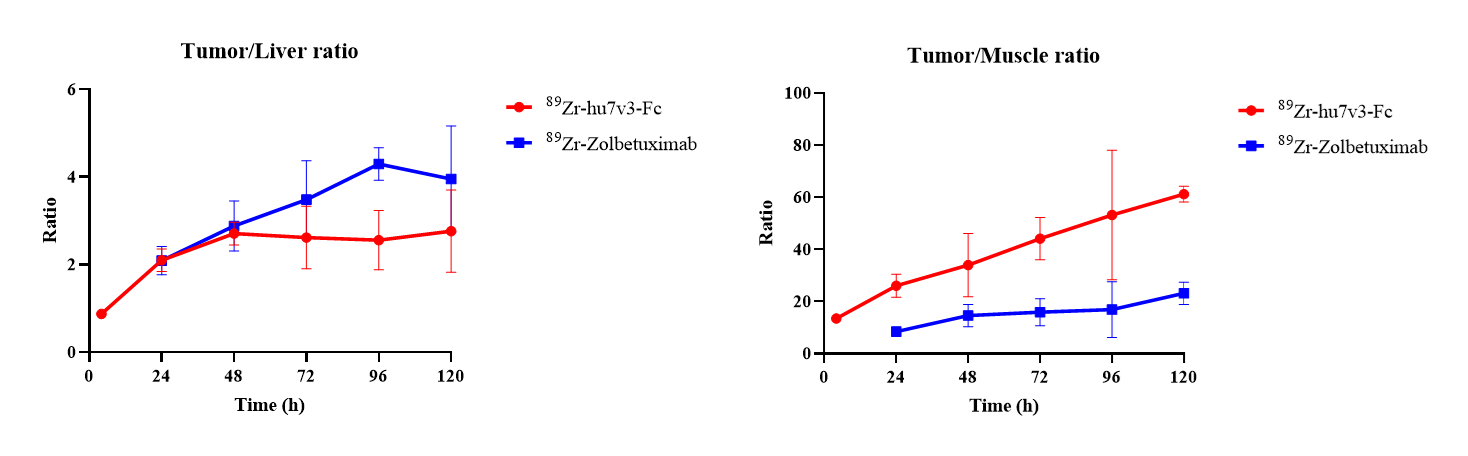

Supplement: Supplementary file 6 [file Image_6.tif]
