# Supplementary material for: Development of a Humanized VHH Based Recombinant Antibody Targeting Claudin 18.2 Positive Cancers
Source: Front Immunol. 2022 Jun 28;13:885424. doi: 10.3389/fimmu.2022.885424 (PMC9273722; doi:10.3389/fimmu.2022.885424)
Supplement: Supplementary file 7 [file Table_1.docx]

**Supplementary Methods and Materials**

**Supplementary Methods**

**Determination of C1q binding to cells opsonized with anti-CLDN18.2 antibody**

C1q (Complement Technology ) was conjugated to biotin using Sulfo-NHS-LC-LC-biotin (Thermo) with a molar ratio 10:1 of biotin to protein. CHO-CLDN18.2-GFP or NUGC-CLDN18.2 cells were suspended in CD02 or RPMI 1640 medium with a cell density of 2×10^6^ cells/ml at 4 ℃. Biotin-C1q was added to a final concentrations of 0-60 μg/ml and hu7v3-Fc, Zolbetuximab analog or hIgG isotype were then added to a final concentration of 100 nM. The reaction mixtures were incubated for 60 minutes at 4 ℃. Cells were then washed twice with PBS, probed with Streptavidin-APC (Southern Biotech) for 30 minutes at 4 ℃. Cells were then washed twice with PBS and analyzed using flow cytometry on a flow cytometer (Agilent).

**BLI analysis to determine the affinity of hu7v3-Fc to CLDN18.2.**

Biolayer interferometry (BLI) assay was used to determine the affinity of hu7v3-Fc to CLDN18.2 based on Octet Platform (RED96e, Fortebio). Briefly, 1 × PBS (pH = 7.4) with 0.01% Tween-20, 0.1% bovine serum albumin (BSA), was used as assay buffer. The hu7v3-Fc was captured *via* AHC Biosensors at a concentration of 10 μg/ml, resulting in a response of 1 nM. Subsequently, the loaded biosensors were equilibrated for 60 s in assay buffer to establish a stable baseline. The association of hu7v3-Fc and CLDN18.2 (100, 50, 25, 12.5, 6.25, 3.125 and 0 nM in assay buffer) was measured for 60 s and the dissociation of them was measured for 120 s in assay buffer. The binding affinity constant K*_D_* values were calculated using 1:1 binding model through global fitting model.

**Legends to Supplementary Figure**

**Supplementary Figure 1. Amino acid sequences.**

DNAMAN was used to generate the alignment. Humanized h-NbBcII10*_FGLA_* and human DP-47 were chosen as reference sequences for humanization. FRs and CDRs are shown. Key amino residues to humanized of FR2 are shaded with gray boxes.

**Supplementary Figure 2. Binding affinity of humanized VHHs-Fc to CLDN18.2 expressing cells by Flow cytometry.**

The analytes were serially diluted, and binding was measured using DyLight 650 conjugated goat anti-human IgG Fc cross-absorbed secondary antibody (Invitrogen).

**Supplementary Figure 3. Binding specificity to CLDN18.2.**

IHC analysis to determine the specificity of hu7v3-Fc to CLDN18.2. Frozen sections of stomach antrum and stomach body were stained with hu7v3-Fc and observed with 400 × magnification.

**Supplementary Figure 4. Binding affinity to CLDN18.2.**

**A and B:** Flow cytometric analysis to determine the affinity of hu7v3-Fc to CLDN18.2. A, mean fluorescence intensity (MFI). B, percentage of positively stained cells.

**C:** BLI analysis to determine the affinity of hu7v3-Fc to CLDN18.2.

**Supplementary Figure 5. Binding affinity to C1q.**

**A:** Flow cytometric analysis to determine the expression level of CLDN18.2 in NUGC4-CLDN18.2 (Up two) and CHO-CLDN18.2-GFP (Bottom two). Cells were stained with hu7v3-Fc (positive) or hIgG isotype control (negative).

**B:** Flow cytometric analysis to determine the binding affinity of C1q to antibody-opsonized cells. Cells were incubated with antibodies at a fixed concentration of 100 nM and biotin-C1q at a concentrations of 0-60 μg/ml.

**Supplementary Figure 6. The tumor to organs ratio.**

Tumor to liver ratio (left) and tumor to muscle ratio (right) calculated from biodistribution study. ^89^Zr-hu7v3-Fc revealed higher tumor/muscle ratio and comparable tumor/liver ratio to ^89^Zr-Zolbetuximab.

**Supplementary Tables**

**Supplementary Table 1** **Antitumor activity in mice implanted with SNU-620 after treatment with hu7v3-Fc and Zolbetuximab analog.**

| **Treatment** | **Tumor Volume (mm^3^)^a^** | **TGI^b^** | **p value(T-test)^c^**  **(%)** |
| --- | --- | --- | --- |
|  | **35^th^ day** | **(%)** |  |
| Vehicle | 1588.6 ± 244.9 | -- | -- |
| hu7v3-Fc (0.3 mg/kg) | 218.0 ± 61.2 | 96.7% | <0.001 |
| hu7v3-Fc (1.0 mg/kg) | 270.5 ± 83.0 | 93.1% | <0.001 |
| hu7v3-Fc (3.0 mg/kg) | 107.9 ± 19.2 | 104.5% | <0.001 |
| Zolbetuximab analog(5.7mg/kg) | 345.0 ± 113.0 | 84.5% | <0.001 |

a. Tumor volume on day 35, Mean ± SEM, n = 8

b. TGI (%) = [1-(T_35_-T_0_)/(V_35_-V_0_)] × 100%

c. *p* (t-test) vs. vehicle.

**Supplementary Table 2** **Antitumor activity in mice implanted with MIA PaCa-2-CLDN18.2 after treatment with hu7v3-Fc and Zolbetuximab analog.**

| **Treatment** | **Tumor Volume (mm^3^)^a^** | **TGI^b^** | **p value(T-test)^c^**  **(%)** |
| --- | --- | --- | --- |
|  | **33^rd^ day** | **(%)** |  |
| Vehicle | 1902.5 ± 208.1 | -- | **--** |
| hu7v3-Fc (0.3 mg/kg) | 630.3 ± 94.5 | 76.3% | <0.001 |
| hu7v3-Fc (1.0 mg/kg) | 335.8 ± 59.9 | 94.0% | <0.001 |
| hu7v3-Fc (3.0 mg/kg) | 388.6 ± 80.1 | 90.9% | <0.001 |
| Zolbetuximab analog(5.7mg/kg) | 1037.4 ± 216.9 | 51.9% | 0.012 |

a. Tumor volume on day 33, Mean ± SEM, n = 8

b. TGI (%) = [1-(T_33_-T_0_)/(V_33_-V_0_)] × 100%

c. *p* (t-test) vs. vehicle.
